# Standards for the diagnosis and management of complex regional pain syndrome: Results of a European Pain Federation task force

**DOI:** 10.1002/ejp.1362

**Published:** 2019-02-18

**Authors:** Andreas Goebel, Chris Barker, Frank Birklein, Florian Brunner, Roberto Casale, Chris Eccleston, E. Eisenberg, Candy S. McCabe, G. Lorimer Moseley, R. Perez, Serge Perrot, Astrid Terkelsen, Ilona Thomassen, Andrzey Zyluk, Chris Wells

**Affiliations:** ^1^ Walton Centre NHS Foundation Trust Liverpool UK; ^2^ Pain Research Institute University of Liverpool Liverpool UK; ^3^ Department of Neurology University of Mainz Mainz Germany; ^4^ Physical Medicine and Rheumatology Balgrist University Hospital Zurich Switzerland; ^5^ Pain Rehabilitation Unit Habilita Hospitals Zingonia di Ciserano Italy; ^6^ Centre for Pain Research The University of Bath Bath Uk; ^7^ Department of Clinical and Health Psychology Ghent University Ghent Belgium; ^8^ European Pain Federation Brussels Belgium; ^9^ Rambam Health Care Campus Institute of Pain Medicine Haifa Israel; ^10^ Florence Nightingale Foundation Clinical Professor of Nursing University of West of England, Bristol & Royal United Hospitals NHS Foundation Trust Bath UK; ^11^ Sansom Institute, University of South Australia Adelade Australia; ^12^ Department of Anaesthesiology VU University Medical Center Amsterdam Netherlands; ^13^ Pain Center Cochin Hospital, Paris Descartes University Paris France; ^14^ Danish Pain Research Center and Department of Neurology Aarhus University Hospital Aarhus Denmark; ^15^ Patiëntenvereniging CRPS Nijmegen The Netherlands; ^16^ Department of General and Hand Surgery Pomeranian Medical University Szczecin Poland

## Abstract

**Background:**

Complex regional pain syndrome is a painful and disabling post‐traumatic primary pain disorder. Acute and chronic complex regional pain syndrome (CRPS) are major clinical challenges. In Europe, progress is hampered by significant heterogeneity in clinical practice. We sought to establish standards for the diagnosis and management of CRPS.

**Methods:**

The European Pain Federation established a pan‐European task force of experts in CRPS who followed a four‐stage consensus challenge process to produce mandatory quality standards worded as grammatically imperative (must‐do) statements.

**Results:**

We developed 17 standards in 8 areas of care. There are 2 standards in diagnosis, 1 in multidisciplinary care, 1 in assessment, 3 for care pathways, 1 in information and education, 4 in pain management, 3 in physical rehabilitation and 2 on distress management. The standards are presented and summarized, and their generation and consequences were discussed. Also presented are domains of practice for which no agreement on a standard could be reached. Areas of research needed to improve the validity and uptake of these standards are discussed.

**Conclusion:**

The European Pain Federation task force present 17 standards of the diagnosis and management of CRPS for use in Europe. These are considered achievable for most countries and aspirational for a minority of countries depending on their healthcare resource and structures.

**Significance:**

This position statement summarizes expert opinion on acceptable standards for CRPS care in Europe.

## 1 INTRODUCTION

Complex regional pain syndrome (CRPS) is a painful and disabling post‐traumatic primary pain disorder affecting, usually, distal limbs (Birklein, Ajit, Goebel, Perez, & Sommer, 2018; Marinus et al., 2011). Signs and symptoms are normally restricted to the affected limb but can spread (van Rijn et al., 2011). The incidence of CRPS may vary between populations (Sandroni, Benrud‐Larson, McClelland, & Low, 2003). In a population‐based European study, the incidence was 20–26/100,000 in the Netherlands (de Mos et al., 2007).

**Figure 1:**
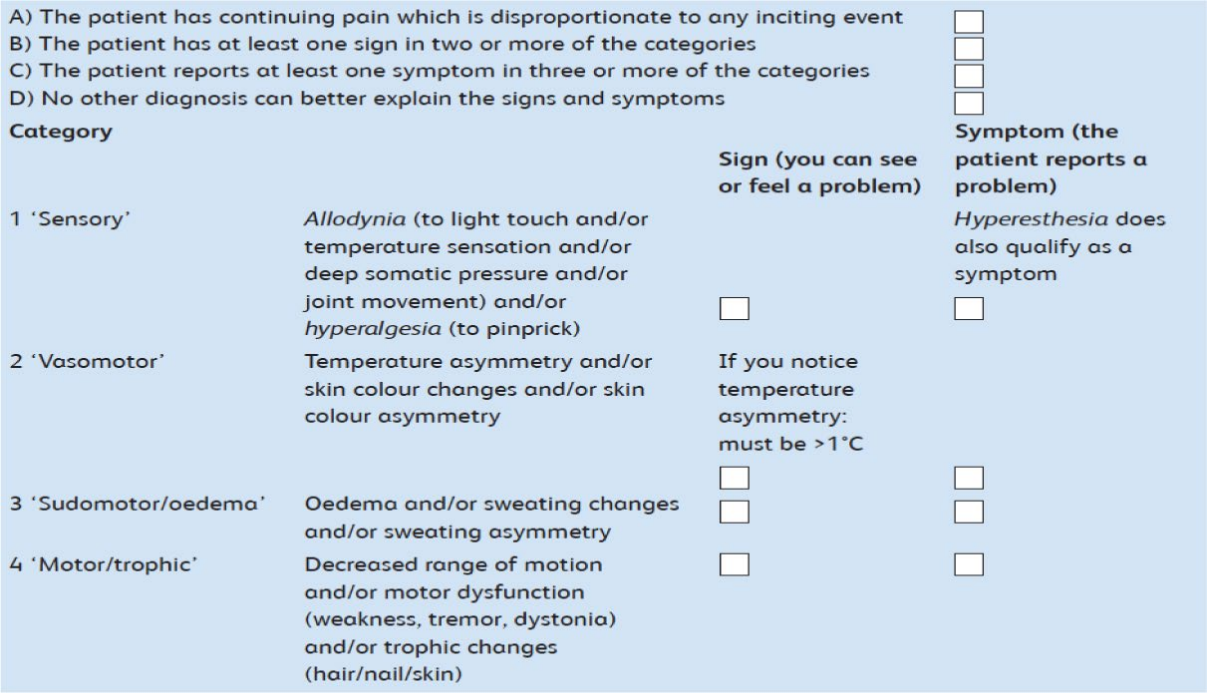
Budapest Diagnostic Criteria for CRPS. *Notes*: (1) If the patient has a lower number of signs or symptoms, or no signs, but signs and/or symptoms cannot be explained by another diagnosis, “CRPS‐NOS” (not otherwise specified) can be diagnosed. This includes patients who had documented CRPS signs/symptoms in the past. (2) If A, B, C and D above are all ticked, please diagnose CRPS. If in doubt, or for confirmation, please refer to your local specialist. (3) Psychological findings, such as anxiety, depression or psychosis, do not preclude the diagnosis of CRPS (3) Distinction between CRPS type 1 (no nerve injury) and CRPS type 2 (major nerve injury) is possible, but has little relevance for treatment. *Explanation of terms*: “Hyperalgesia” is when a normally painful sensation (e.g., from a pinprick) is more painful than normal; “allodynia” is when a normally not painful sensation (e.g., from touching the skin) is now painful; and “hyperaesthesia” is when the skin is more sensitive to a sensation than normal. *A special feature in CRPS*: In category 4, the decreased range of motion/weakness is not always due to pain. It is also not necessarily due to nerve damage or a joint or skin problem. Some patients’ experience of an inability to move their limb may be due to yet poorly understood, disturbed motor coordination which can be reversible. A helpful question to assess this feature is: “If I had a magic wand to take your pain away, could you then move your… (e.g., fingers)?” Many patients will answer with “no” to that question. *Unusual CRPS*: Around 5% of patients cannot recall a specific trauma or may report that their CRPS developed with an everyday activity such as walking or typewriting. In very few people, CRPS can have a bilateral onset. In some patients, CRPS can spread to involve other limbs. Around 15% of CRPS cases do not improve after 2 years. It is appropriate to make the diagnosis of CRPS in these unusual cases

The clinical presentations of CRPS vary enormously between patients (Figure 1). For example, the affected limb may appear hot and red, or cold and blue; these symptoms and signs can also fluctuate in any single patient over time. Patients often also report disordered spatial awareness, and bodily and limb agency distortions (Lewis, Kersten, McCabe, McPherson, & Blake, 2007).

The aetiology of CRPS is likely multifactorial (Birklein et al., 2018). It is thought to be pathological not psychopathological in origin (Beerthuizen et al., 2012; Beerthuizen, van 't Spijker, Huygen, Klein, & de Wit, 2009). Most patients will improve over time (de Mos et al., 2009; Zyluk, 1998), although appropriate management very likely hastens recovery (Gillespie, Cowell, Cheung, & Brown, 2016). However, full recovery is less common, and many patients will be left with varying degrees of persistent pain and functional impairment (Bean, Johnson, Heiss‐Dunlop, & Kydd, 2016). For some people, CRPS may become a long‐lasting, highly disabling and distressing chronic pain condition. The costs of CRPS are significant, at a personal, familial and societal level (Kemler & Furnee, 2002; van Velzen et al., 2014).

In 2016, the European Pain Federation convened a CRPS Task Force to support the development of best care for these patients through Europe. The Task Force members were CRPS experts with geographical and professional representation within Europe and a patient representative. As its first objective, the Task Force was asked to develop *standards* that could guide minimally acceptable levels of CRPS care applicable across a diversity of healthcare structures and economies within Europe.

Some European countries have developed their own *guidelines* for CRPS care (Birklein, Humm et al., 2018; Ceruso et al.., 2014; Goebel et al., 2018; Perez et al., 2014); however, any adoption within additional countries is often impeded by differences in healthcare economics and structures. The application of *standards* can go some way to establish a primary common position.

**Figure 2:**
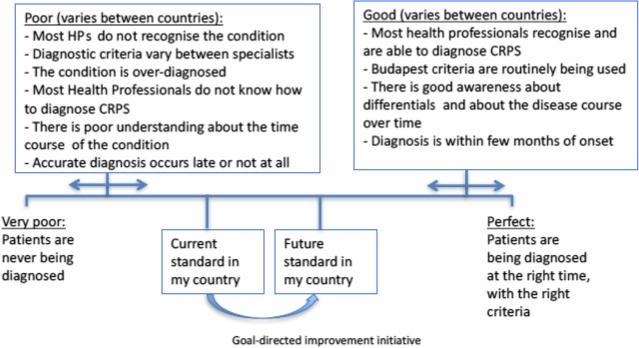
The European Task Force dynamic diagnostic standard quality framework for CRPS. HP: health professional

We recognize that terms such as “standards,” “guidelines,” “policy” and “procedure” are often used interchangeably, and currently, there is no internationally agreed definition for the term “standards” as applied to health care. For our purposes, we considered the UK Faculty of Pain Medicine interpretation of standards, as applied to pain: “Standards must be followed. Standards aim to represent *current best practice* in pain management as published in relevant literature and/or agreed by a body of experts” (Grady & al., 2015, p. 8). Notably, standards can change over time (Figure 2). Standards can act as a benchmark, but can also be utilized as a tool for healthcare professionals, commissioners and policymakers in the identification and appropriate allocation of resources.

## 2 METHODS

Our development process followed that outlined by the UK National Institute of Clinical Excellence (NICE, see Supporting Information Appendix S1). A patient‐member (IT) provided service user perspectives. The CRPS standards were derived through discussion and a process of consolidation and challenge which had four stages: First, we took account of the evidence from recently published systematic reviews (Duong, Bravo, Todd, & Finlayson, 2018; O'Connell, Wand, McAuley, Marston, & Moseley, 2013). Second, a draft document outlining the domains of practice and the likely areas of difference was produced and discussed in e‐mail and telephone discussions from November 2016 to May 2017. Third, we convened a one‐day face‐to‐face meeting in June 2017. The focus of the meeting was to seek agreement among the members of the Task Force on the areas of practice. A “challenge” process was developed in which we drafted the standards as grammatically imperative (must‐do) statements. This presentation of each standard of care as mandatory was useful because it forced members to think about exceptional cases or alternatives. Finally, a draft document of standards was then drafted. Each member of the group had one more opportunity to veto any highly contentious area and suggest further changes. No veto was enacted.

The resulting standards were considered achievable for most countries and aspirational for a minority of countries depending on their healthcare resource and structures (Eccleston, Wells, & Morlion, 2018).

## 3 RESULTS

We developed 17 *standards*, highlighted in italics, in 8 areas of care. There are 2 *standards* in diagnosis, 1 in multidisciplinarity, 1 in assessment, 3 in care pathways, 1 in information and education, 4 in pain management, 3 in physical rehabilitation and 2 in distress management.

### 3.1 The diagnosis of complex regional pain syndrome

Complex regional pain syndrome is diagnosed according to the “New IASP Criteria” (sensitivity: 0.99; specificity: 0.68 for the “clinical” criteria) (Harden & Bruehl, 2005; Harden et al., 2010) which are sometimes also referred to as the “Budapest criteria” (Figure 1).

The use of these criteria requires some degree of prior belief that the condition is likely to be CRPS, that is, the patient has a regional affection distally in extremities, not corresponding to a nerve innervation territory. As an exception, the rare subtype of CRPS II after nerve injury can sometimes correspond to the injured nerve's innervation territory. These criteria stipulate that CRPS is a diagnosis of exclusion, and alternative (“differential”) diagnoses are provided in Box 1.

BOX 1 **Possible differential diagnoses**

1

1. Local pathology: Distortion, fracture, pseudoarthrosis, arthrosis, inflammation (cellulitis, myositis, vasculitis, arthritis, osteomyelitis and fasciitis), compartment syndrome and immobilization‐induced symptoms. Persistent defects after limb injury: osteoarthritis developing after joint fractures; myofascial pain due to changed (protective) movement patterns
2. Affection of arteries, veins or lymphatics, for example traumatic vasospasm, vasculitis, arterial insufficiency, thrombosis, Raynaud's syndrome, thromboangiitis obliterans (Buerger's syndrome), lymphedema and secondary erythromelalgia.
3. Connective tissue disorder
4. Central lesion, for example spinal tumour
5. Peripheral nervous system lesion (nerve compression, cervico‐brachial or lumbo‐sacral plexus affection, acute sensory polyneuropathy, (poly‐)neuritis, autoimmune (e.g., posttraumatic vasculitis) and infectious (e.g., borreliosis))
6. Malignancy (Pancoast tumour/paraneoplastic syndrome/occult malignancy)
7. Factitious disorder

Particular awareness about differential diagnosis is advised in spontaneously developing CRPS (no trauma, about 5% of cases), when the involvement is a proximal part of the limb, such as the shoulder, or when there is primary involvement of more than one limb.

Uncertainty about the diagnosis can be distressing to patients and may lead to inappropriate treatment. European countries differ in their current standards about the timely manner of diagnosing CRPS; however, each country is better than the worst situation: where patients are never being diagnosed (Figure 2).

Improvements in diagnostic standards are possible and desirable through information and training of healthcare professionals and patients. For example, in Switzerland an information leaflet about CRPS was sent to all practising medical doctors in the country, and there is consensus that awareness has improved (SUVA, 2013).

While there are “perfect” diagnostic standards, it is important to establish realistic, country‐related next goals and consequently identify which steps that aim to improve current standards will help to achieve these goals (see Figure 2)—this process can later be repeated as appropriate. The use of a diagnostic checklist is helpful, as shown in Figure 1. The European Pain Federation task force members recognize the challenges about the future development of the CRPS Budapest criteria (Table 2); these challenges include, among others, the diagnostic approach to a small number of patients diagnosed according to Budapest criteria, who over time lose some of their CRPS signs such as swelling, but have unchanged pain. These patients are currently labelled as “CRPS—not otherwise specified” (CRPS‐NOS, Table 2), which has sometimes led to challenges with the reimbursement of therapies, or in the context of insurance‐ and medico‐legal proceedings, and a better solution may be required.

***Standard*** 1: “Budapest” diagnostic criteria for CRPS must be used, as they provide acceptable sensitivity and specificity.

***Standard*** 2: Diagnosing CRPS does not require diagnostic tests, except to exclude other diagnoses.

It is worth noting that different opinions existed within the Task Force regarding the usefulness of three‐phase bone scintigraphy or magnetic resonance imaging for the diagnosis of CRPS, with some members considering these techniques useful, and the majority not. There was agreement that existing tests do not reflect pathognomonic parameters.

### 3.2 The management and referral of patients with CRPS

**Figure 3:**
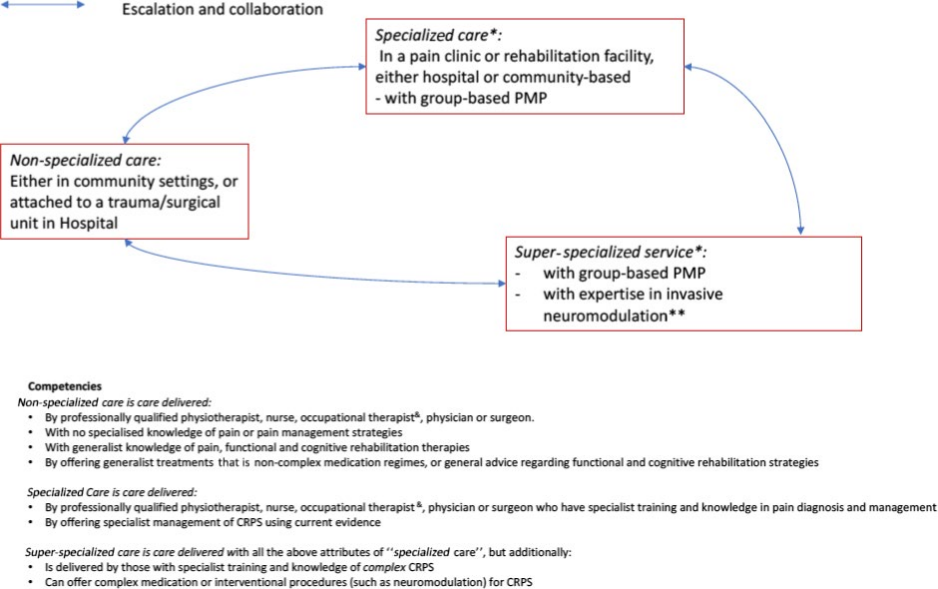
Services and competencies. PMP = multidisciplinary pain management programme integrating psychological care and functional rehabilitation; ^&^additionally “Hand Therapists” in some European Countries, *note, some pain clinics and rehabilitation facilities do not provide group‐based PMP, whereas others additionally provide “super‐specialized” services; **neuromodulation is listed to highlight the care structure within which it is delivered; some centres will not provide this service

Standard 3: The management of mild (mild pain and mild disability) CRPS may not require a multi‐professional team; however, the degree of severity and complexity of CRPS must dictate the need for appropriately matched multi‐professional care (for details, see section care structure and Figure 3).

Standard 4: Patients diagnosed with CRPS must be appropriately assessed; this assessment must establish any triggering cause of their CRPS, their pain intensity and the interference their pain causes on their function, their activities of daily living, participation in other activities, quality of life, sleep and mood.

Most patients have short‐lasting CRPS which may improve within a few months, even without treatment (Zyluk, 1998), so that these patients are best treated in non‐specialized care, provided by healthcare professionals who have had standard training within their discipline (e.g., physiotherapist and general practitioner—see Figure 3); early treatment is highly likely to shorten the time of suffering for many patients (Gillespie et al., 2016).

Standard 5: Referral to specialized care must be initiated for those patients who do not have clearly reducing pain and improving function, within 2 months of commencing treatment for their CRPS, despite good patient engagement in rehabilitation.

There is consensus that the best exact time may vary somewhat between patients, but that 2 months is a reasonable guide.

Standard 6: Referral to super‐specialized care must be initiated for the small number of patients with complications such as CRPS spread, fixed dystonia, myoclonus, skin ulcerations or infections or malignant oedema in the affected limb, and those with extreme psychological distress.

Referral to super‐specialized care may also be appropriate for patients which are not improving in specialized services: (a) for additional expertise in treating this rare patient group and (b) for consideration of interventions not available in specialized care (Figure 3).

There was no consensus about the best names for these three types of services, although most Task Force members considered the current wording in standard 6 to be acceptable. There is agreement that other wordings may be substituted as is nationally or locally appropriate.

Treating healthcare professionals should be aware of appropriate specialized care services and any services with specific expertise and interest in the management of CRPS nationally (“super‐specialized” care facilities), Figure 3.

Standard 7: Specialized care facilities must provide advanced treatments for CRPS including multidisciplinary psychologically informed rehabilitative pain management programmes (PMP). If they do not provide these treatments, then they must refer for these treatments, if needed, to other specialized care facilities, or to super‐specialized care facilities (Figure 2).

We propose that specialized care facilities (Figure 3), who wish to establish quality indicators about their regional CRPS pathway, should in the first instance establish an internal registry of CRPS cases seen. Since the incidence of CRPS in Europe (20–26/100,000) is known, such a registry may support professionals to estimate whether those patients in their region who are in need of their service do in fact reach them. The registry, once established, can also serve as a basis for additional quality improvement efforts.

Each Chapter of the European Pain Federation should institute an appropriate treatment guideline for CRPS that is valid for the circumstances in that country, even if this is adapted from existing guidelines in other countries. Production of lay audience‐appropriate versions should be considered.

### 3.3 Prevention

Early, appropriate rehabilitation treatment post‐trauma may prevent the development of CRPS; however, more data are needed to fully understand its impact (Gillespie et al., 2016). A high pain score one week after trauma may indicate a “fracture at risk” (Moseley et al., 2014) and thus identify patients who benefit most from preventative early rehabilitation.

There is conflicting evidence about the value of using vitamin C after distal radius fracture to prevent the development of CRPS. There is also very preliminary evidence about the value of steroids to prevent a prolonged course of CRPS after very early CRPS has been diagnosed. More studies are needed before recommendations can be given.

The Task Force decided that there is insufficient evidence for or against any methods of prevention to allow for a standard to be written.

### 3.4 Patient information and education

Standard 8: Patients, and where appropriate their relatives and carers must receive adequate information soon after diagnosis on (a) CRPS, (b) its causation (including the limits of current scientific knowledge), (c) its natural course, (d) signs and symptoms, including body perception abnormalities, (e) typical outcomes and (f) treatment options. Provision of information is by all therapeutic disciplines and must be repeated as appropriate.

Emphasis should be put on the goals of treatment and on the patient's active involvement in the treatment plan. The typically benign prognosis should be emphasized.

Information is available from various sources (e.g., ARUK, 2016; Birklein, Humm, et al., 2018; Ceruso et al., 2014; Crpsvereniging, 2018; Goebel et al., 2018; Perez et al., 2014).

### 3.5 Pain management—medication and procedures

Standard 9: Patients must have access to pharmacological treatments that are believed to be effective in CRPS. Appropriate pain medication treatments are considered broadly similar with those for neuropathic pains, although high‐quality studies in CRPS are not available (Duong et al., 2018). All patients with CRPS must receive a pain treatment plan consistent with any geographically relevant guidelines.

Treatment with bisphosphonates and/or steroids has also been considered. However, the Task Force members did not reach agreement about the evidence for or against their efficacy and safety.

Standard 10: Efforts to achieve pain control must be accompanied by a tailored rehabilitation plan

Standard 11: Medications aimed at pain relief may not be effective in CRPS, while causing important adverse effects; therefore, stopping rules should be established and a medication reduction plan must be in place if on balance continuation is not warranted.

Standard 12: CRPS assessment (see above) must be repeated as appropriate, because both the natural development of the disease and of treatment may change the clinical picture over time.

Some patients who have not responded to other treatments may be considered for invasive neuromodulation and should be referred for assessment.

### 3.6 Physical and vocational rehabilitation

In partnership with the patient, appropriate, generally gentle, graded exercises in the presence of pain should be advised upon by a trained healthcare professional; this is essential as to give the best chance of a good outcome and minimize distress. Immobilization of the CRPS limb should be avoided wherever possible. (Gillespie et al., 2016; Oerlemans, Oostendorp, de Boot, & Goris, 1999/10).

Standard 13: Patient's limb function, overall function and activity participation, including in the home and at work or school, must be assessed early and repeatedly as appropriate. Patients should have access to vocational rehabilitation (as relevant).

Standard 14: Patients with CRPS must have access to rehabilitation treatment, delivered by physiotherapists and/or occupational therapists, as early as possible in their treatment pathway.

This may shorten the early disease course and preserve limb function. In some European countries, these treatments are guided by medical doctors, including rehabilitation specialists, general practitioners or others.

Standard 15: Physiotherapists and occupational therapists must have access to training in basic methods of pain rehabilitation and CRPS rehabilitation

### 3.7 Identifying and treating distress

Standard 16: Patients must be screened for distress including depression, anxiety, post‐traumatic stress, pain‐related fear and avoidance. This must be repeated where appropriate (Bean, Johnson, Heiss‐Dunlop, Lee, & Kydd, 2015).

Standard 17: Where required, patients must have access to evidence‐based psychological treatment

### 3.8 Long‐term care

Some patients will continue to experience impediments to their quality of life even after appropriate treatment has been completed. These impediments either are due to ongoing consequences of CRPS even though the condition has improved (about 40% of all patients), or are caused by unresolved CRPS (about 15%‐20%; de Mos et al., 2009). Particularly, the latter group may benefit from the offer of a long‐term management plan, mainly aiming to maximize support for self‐management. Long‐term management is ideally initiated through specialized or super‐specialized services and may include referral back to these services if CRPS‐specific symptoms change (Figure 3); an example is described here (RUHNHSFT, 2016).

## 4 DISCUSSION

We here present 17 standards for the diagnosis and management of CRPS for consideration of adoption in Europe. They are summarized in Table 1.

**Table 1:** European Pain Federation standards for the diagnosis and management of complex regional pain syndrome

Diagnosis	Standard 1	“Budapest” diagnostic criteria for CRPS must be used, as they provide acceptable sensitivity and specificity.
	Standard 2	Diagnosing CRPS does not require diagnostic tests, except to exclude other diagnoses.
Management and Referral	Standard 3	The management of mild (mild pain and mild disability) CRPS may not require a multi‐professional team; however, the degree of severity and complexity of CRPS must dictate the need for appropriately matched multi‐professional care (for details, see section care structure and Figure 3).
	Standard 4	Patients diagnosed with CRPS must be appropriately assessed; this assessment must establish any triggering cause of their CRPS, their pain intensity and the interference their pain causes on their function, their activities of daily living, participation in other activities, quality of life, sleep and mood.
	Standard 5	Referral to specialized care must be initiated for those patients who do not have clearly reducing pain and improving function within 2 months of commencing treatment for their CRPS despite good patient engagement in rehabilitation.
	Standard 6	Referral to super‐specialized care must be initiated for the small number of patients with complications such as CRPS spread, fixed dystonia, myoclonus, skin ulcerations or infections or malignant oedema in the affected limb, and those with extreme psychological distress.
	Standard 7	Specialized care facilities must provide advanced treatments for CRPS including multidisciplinary psychologically informed rehabilitative pain management programmes (PMP). If they do not provide these treatments, then they must refer for these treatments, if needed, to other specialized care facilities, or to super‐specialized care facilities (Figure 3).
Prevention	None	No Standards were considered as having sufficient support to recommend as mandatory.
Information and Education	Standard 8	Patients and where appropriate their relatives and carers must receive adequate information soon after diagnosis on (a) CRPS, (b) its causation (including the limits of current scientific knowledge), (c) its natural course, (d) signs and symptoms, including body perception abnormalities, (e) typical outcomes and (f) treatment options. Provision of information is by all therapeutic disciplines and must be repeated as appropriate.
Pain Management	Standard 9	Patients must have access to pharmacological treatments that are believed to be effective in CRPS. Appropriate pain medication treatments are considered broadly similar with those for neuropathic pains, although high‐quality studies in CRPS are not available (Duong et al., 2018). All patients with CRPS must receive a pain treatment plan consistent with any geographically relevant guidelines.
	Standard 10	Efforts to achieve pain control must be accompanied by a tailored rehabilitation plan.
	Standard 11	Medications aiming at pain relief may not be effective in CRPS, while causing important side effects; therefore, stopping rules should be established and a medication reduction plan must be in place if on balance continuation is not warranted.
	Standard 12	CRPS assessment (see above) must be repeated as appropriate, because both the natural development of the disease and of the treatment may change the clinical picture over time.
Physical and Vocational Rehabilitation	Standard 13	Patient's limb function, overall function and activity participation, including in the home and at work or school, must be assessed early and repeatedly as appropriate. Patients should have access to vocational rehabilitation (as relevant).
	Standard 14	Patients with CRPS must have access to rehabilitation treatment, delivered by physiotherapists and/or occupational therapists, as early as possible in their treatment pathway.
	Standard 15	Physiotherapists and occupational therapists must have access to training in basic methods of pain rehabilitation and CRPS rehabilitation.
Identifying and Treating Distress	Standard 16	Patients must be screened for distress including depression, anxiety, post‐traumatic stress, pain‐related fear and avoidance. This must be repeated where appropriate.
	Standard 16	Where required, patients must have access to evidence‐based psychological treatment.
Long‐term Care	None	No standards were considered as having sufficient support to recommend as mandatory.
Version January 2019		

These standards can be considered best practice in CRPS as supported by expert and patient agreement. We followed a method that focused on evidence review but which prioritized the production of a series of mandatory statements of optimal clinical practice that could be followed in the majority of the 37 countries who are members of the European Pain Federation. We deliberately avoided statements of optional, desirable or aspirational practice, focusing on what was considered achievable by most.

There are a number of limitations to our approach that should be taken into account. First, we did not canvas all clinicians working in this field across all of the 37 countries, or managers, politicians or other non‐healthcare stakeholders. We focused instead on an expert group supported by a patient representative. It is possible that different experts would have produced different standards. This was a deliberate decision on our behalf as we needed to set a first list of expert‐driven standards from which to build. Second, we did not produce a series of evidence syntheses (e.g., meta‐analytic review of efficacy or review of assessment tools). We judged that such an effort would be resource‐heavy and unlikely to yield any clarity due to the well‐documented absence of primary research into this orphan disease. Instead, we relied on the extant literature which is well known to the group. Third, our decision to craft standards as mandatory meant that the heterogeneity of different views and nuanced opinions was not reported. Presented only is the result.

The standards and their production have clinical, research and policy implications.

1. Clinical implications: the next step is to share the standards with Federation members, which has a number of challenges. First, language translation of the standards is necessary. Second, we need to survey clinicians for current practice as it relates to the standards to establish a baseline of common clinical practice.
2. Research implications: there is no standard that could not benefit from further study. And there are two areas where we were unable to set standards of care. The group considered that a priority for research was to better understand the heterogeneity of presentation within the current broad category of CRPS. For example, there is a need to differentiate between an early and late presentation. There is a need to look at sex and age differences. And, there is a need to look at CRPS in the context of comorbidities. There are also challenges to the Budapest criteria which, summarized in Table 2, need urgent attention.
3. Policy implications: these standards are the first step in a process. Standards are essentially a tool to improve practice, but practice only improves if they are used. We next need to understand the barriers to their implementation, whether they are resource, educational, legislative or organizational. We propose that a CRPS pain champion be appointed by each of the 37 national pain chapters, who can guide development and be a point of contact for this work.

**Table 2:** Challenges for the future development of CRPS Budapest criteria that arise from the 17 Standards

Challenge 1	How should we deal with “CRPS‐like conditions” fulfilling only some diagnostic criteria (from the start—never fulfilled Budapest diagnosis)?
Challenge 2	How shall we term those cases of CRPS which initially clearly conformed with Budapest criteria, but which have now very few signs not conforming with Budapest criteria, but ongoing pain. This includes cases, where that pain is as strong as initially, and (more often) other cases, where the pain is improved, but stable and still problematic to the patient's quality of life. We recognize that these cases are rare, since sensory and motor signs providing the basis for the Budapest diagnosis are almost always present. Where the diagnosis of CRPS was correctly made, and documented in the past, might these cases be termed, for example, “partially recovered,” or “sequelae”?
Challenge 3	How can we better clarify the specificity of the Budapest diagnosis outside neuropathic pain settings?

Finally, we recognize that these standards are open to change and should be reviewed regularly. In particular, we need to take account of national standards, practice reviews, guidance and guidelines, either from individual pain societies or those in rehabilitation, neurology or other therapy areas. We need also to be mindful of non‐European work that could influence these standards, including any new and emerging evidence. We have therefore agreed with the European Pain Federation to review these standards five years from their date of publication.

## CONFLICT OF INTEREST

None Declared. GLM receives royalties for books on pain, rehabilitation and CRPS, and speaker fees for lectures on pain, rehabilitation and CRPS. He has received support from Pfizer; Grunenthal; Workers’ Compensation Boards in Australia, Europe and North America; Arsenal Football Club; Port Adelaide Football Club; and the International Olympic Committee.
