# Supplementary material for: Standards for the diagnosis and management of complex regional pain syndrome: Results of a European Pain Federation task force
Source: Eur J Pain. 2019 Feb 18;23(4):641–51. doi: 10.1002/ejp.1362 (PMC6593444; doi:10.1002/ejp.1362)
Supplement: Supplementary file 1 [file EJP-23-641-s001.docx]

**Appendix 1:** National Institute for clinical excellence (NICE) quality standard process guide (page 16: <https://www.nice.org.uk/media/default/Standards-and-indicators/Quality-standards/Quality-standards-process-guide-April-2014.pdf>)


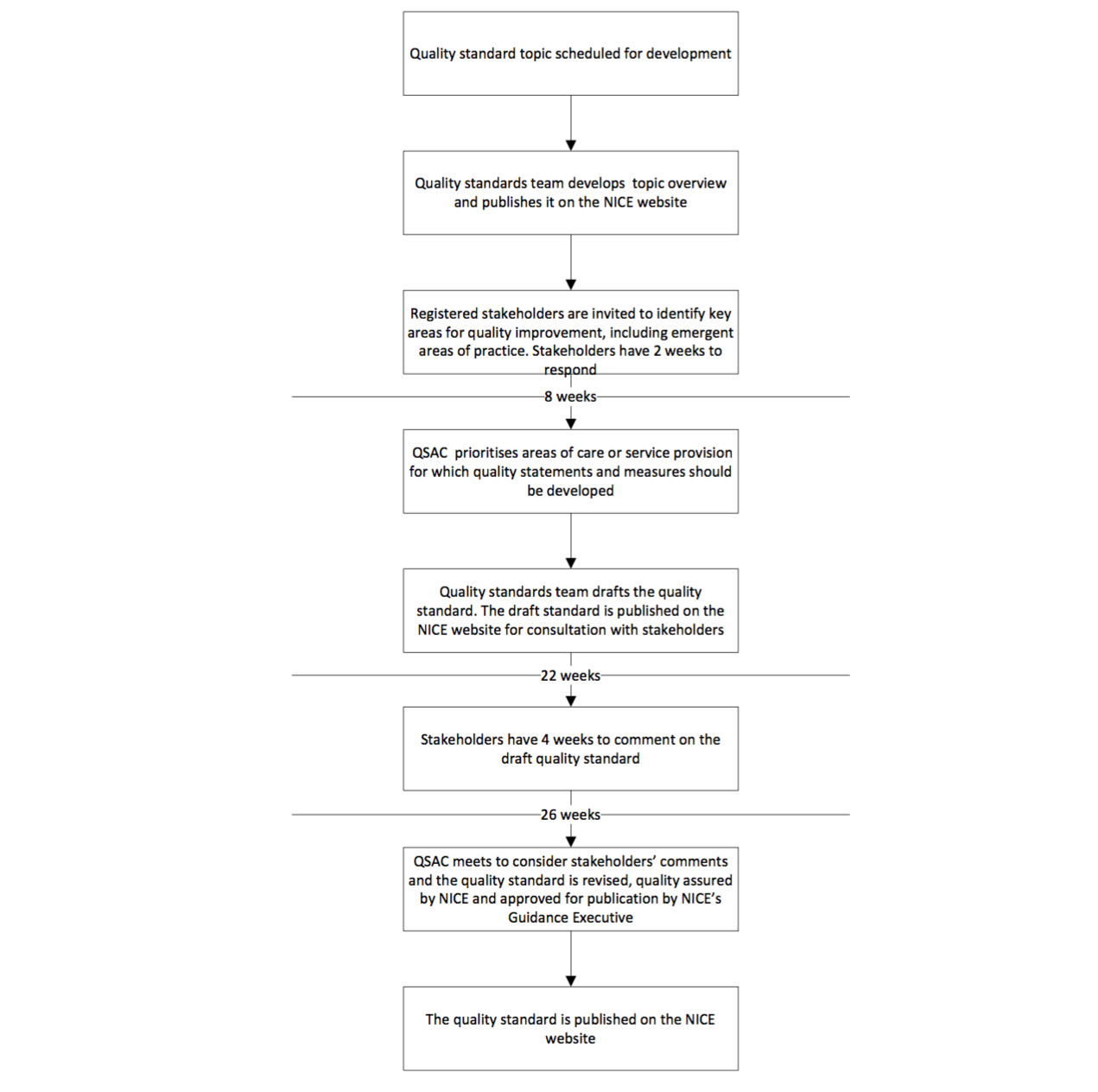


**How does the European CRPS standards development approach compare to this outline guide:**

- Quality standards team: AG and CB, President European Pain Federation
- Stakeholders: European experts and patient representative
- Quality standards advisory committee (QSAC): European Pain Federation Board and Chair of Research (CE)
- Stakeholder engagement though preliminary work: iterative development of separate drafts for CRPS diagnosis and management through e-mail communication.
- Stakeholder meeting to agree key areas & work though disparities given European variance in healthcare structure & practice
- Development of document & dissemination for comments
- QSAC review and revision, with request for some adaptations
- Stakeholder finalization through another iterative electronic process
- Publication
